# Non‐Invasive Hemodynamic Monitoring System Integrating Spectrometry, Photoplethysmography, and Arterial Pressure Measurement Capabilities

**DOI:** 10.1002/advs.202310022

**Published:** 2024-04-22

**Authors:** Jukka‐Pekka Sirkiä, Tuukka Panula, Matti Kaisti

**Affiliations:** ^1^ Department of Computing University of Turku Vesilinnantie 5 Turku 20500 Finland

**Keywords:** blood pressure, hemodynamics, multi‐wavelength photoplethysmography, photoplethysmography, spectrometry

## Abstract

Minimally invasive and non‐invasive hemodynamic monitoring technologies have recently gained more attention, driven by technological advances and the inherent risk of complications in invasive techniques. In this article, an experimental non‐invasive system is presented that effectively combines the capabilities of spectrometry, photoplethysmography (PPG), and arterial pressure measurement. Both time‐ and wavelength‐resolved optical signals from the fingertip are measured under external pressure, which gradually increased above the level of systolic blood pressure. The optical channels measured at 434–731 nm divided into three groups separated by a group of channels with wavelengths approximately between 590 and 630 nm. This group of channels, labeled transition band, is characterized by abrupt changes resulting from a decrease in the absorption coefficient of whole blood. External pressure levels of maximum pulsation showed that shorter wavelengths (<590 nm) probe superficial low‐pressure blood vessels, whereas longer wavelengths (>630 nm) probe high‐pressure arteries. The results on perfusion indices and DC component level changes showed clear differences between the optical channels, further highlighting the importance of wavelength selection in optical hemodynamic monitoring systems. Altogether, the results demonstrated that the integrated system presented has the potential to extract new hemodynamic information simultaneously from macrocirculation to microcirculation.

## Graphical abstract

A system combining the capabilities of spectrometry, photoplethysmography, and arterial pressure measurement is demonstrated to extract hemodynamic parameters from the fingertip. The optical channels effectively split into three groups, representing: i) the smallest blood vessels (microcirculation), ii) an abrupt transition band at the wavelength range where the absorption coefficient of whole blood decreases, and iii) large arteries.

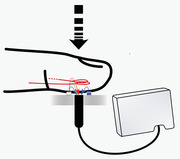

## 1 Introduction

Hemodynamic parameters provide information on the dynamic properties of blood flow within the circulatory system. Such parameters are related to, for example, blood pressure (BP), cardiac output, vascular resistance, and arterial stiffness. Measurement of hemodynamic parameters is an essential part of clinical practice,^[^ ^1^, ^2^ ^]^ especially in critical care and disease diagnosis. Measurement techniques vary from invasive to minimally, or even non‐invasive techniques. The latter two have attracted attention in recent years due to the inherent risk of complications in invasive techniques, but also due to technological development.^[^ ^1^, ^3^, ^4^, ^5^ ^]^

There are several non‐invasive techniques to measure hemodynamic parameters. These include visual observation of capillary refill time, pressure sensing using a cuff or a tonometer, photoplethysmography (PPG), laser Doppler flowmetry, near‐infrared spectroscopy (NIRS), volume clamping, bioimpedance, bioreactance, pulse wave analysis, and pulse wave transit time.^[^ ^1^, ^2^, ^6^ ^]^ Most of these techniques are based on a single sensor modality and thus can only provide a rather limited amount of information. In some cases, a technique can be combined with a method to alter the measured signals to provide more information, for example, in reactive hyperemia measurement where a pneumatic or NIRS sensor is combined with a brachial cuff. Volume‐clamp is a notable exception that combines different sensor modalities together to measure continuous BP.

In this study, we present a non‐invasive system that combines the capabilities of BP measurement, spectrometry, and PPG. Typically, these techniques are used separately: blood pressure measurement using a branchial/wrist/finger cuff, spectrometry using a NIRS system, and PPG in the form of a pulse oximeter for peripheral blood oxygen saturation and heart rate monitoring. However, our system integrates a time‐synchronized visible light ‐ near‐infrared (NIR) spectrometer with an actuator to extract: i) arterial pressure signal, ii) time‐resolved spectra, and iii) high‐resolution multi‐wavelength PPG signals. Thus, the system has potential to provide a rich set of hemodynamic parameters, as it is capable of measuring optical signals as functions of both external pressure and wavelength.

We demonstrate the system in measurements where the level of external pressure gradually increases above systolic BP (SBP). High‐spectral‐resolution PPG signal data is extracted from the spectral data and studied from the perspective of maximum oscillometric pressure (MOP), perfusion index (PI), DC component level, AC component correlation, and area under the low‐pass filtered PPG signal (AUC_lp_). Of these, MOP, PI, and DC component level are parameters that contain hemodynamic information. MOP is related to the mean BP, and is thus an important hemodynamic parameter. The PI is a rather complex parameter, especially affected by vascular tone and stroke volume, and has in recent years attracted renewed research interest,^[^ ^7^ ^]^ with, for example, low values having been associated with severe postoperative complications or death in acute high‐risk surgical patients.^[^ ^8^ ^]^ The DC component level is associated with average blood volume along with the optical properties of tissues and is affected by, for example, vasomotor activity.^[^ ^9^ ^]^ Finally, AC component correlations and AUC_lp_ reveal information about the origins of the different PPG signals.

## 2 Related Work

PPG is a widely used technique to measure cardiovascular activity from the skin using a simple optical sensor that detects volumetric changes of blood within the tissue.^[^ ^9^ ^]^ Variations in blood volume are detected by measuring light absorption using a light source, typically a light‐emitting diode (LED), and a photodetector. A typical application of PPG is the pulse oximeter that utilizes two different wavelengths (which are absorbed differently by oxygenated and deoxygenated hemoglobin) to measure peripheral oxygen saturation, in addition to measuring heart rate. PPG is also a promising technique to measure other hemodynamic parameters such as PI, BP related pulse transit time (PTT)^[^ ^6^ ^]^ and arterial stiffness.^[^ ^10^ ^]^

In recent years, researchers have increasingly studied the technique of using several wavelengths of light simultaneously to extract new hemodynamic information. These include parameters such as arteriolar‐PTT‐based BP,^[^ ^11^, ^12^ ^]^ systemic vascular resistance,^[^ ^13^ ^]^ penetration depth‐dependent PPG,^[^ ^14^ ^]^ and depth‐resolved BP and vasomotor activity.^[^ ^15^ ^]^ Such multi‐wavelength PPG (MWPPG) systems use a photodiode and several time‐multiplexed LEDs. The theoretical foundation behind the technology is based on: i) the penetration ability of light into the skin depends on the wavelength^[^ ^16^, ^17^ ^]^ and ii) the organization of the cutaneous vasculature is such that deeply buried large arteries responsible for blood conduction branch out to blood flow regulating arterioles that themselves branch out to surrounding tissue maintaining capillaries.^[^ ^18^ ^]^ Therefore, MWPPG can obtain hemodynamic information from the different blood vessels buried within the skin.^[^ ^19^, ^20^, ^21^, ^22^ ^]^

Spectroscopy studies the interaction of electromagnetic radiation with matter as a function of wavelength. NIRS is a well‐known application of spectroscopy in the field of physiological monitoring, where NIR radiation is used to observe changes in tissue oxygenation.^[^ ^23^, ^24^ ^]^ In addition to oxygen saturation, spectral data can also provide information on blood components, such as hemoglobin and bilirubin, when the spectra are processed, for example, with the help of a neural network.^[^ ^25^ ^]^ An alternative approach to non‐invasive measurement of blood components is the dynamic spectrum technique, where a spectrometer is used to record time‐resolved spectra from which MWPPG signals, intensity values as a function of time and wavelength, are extracted from a certain part of the visible and NIR part of the electromagnetic spectrum.^[^ ^26^, ^27^, ^28^ ^]^ Thus, a spectrometer can obtain the same information as a conventional MWPPG system by inverting the sensing setup: instead of multiple LEDs and a wideband photodiode, a wideband light source (e.g., white LED) and a spectrometric detector are used. This leads to significantly better spectral resolution (potentially hundreds of channels versus 3–5 in a typical MWPPG sensor). Recent advances have also led to miniaturized spectrometer‐on‐a‐chip solutions,^[^ ^29^, ^30^, ^31^ ^]^ although with a more limited spectral resolution compared to a proper spectrometer (e.g., 11 in ref. [31]).

## 3 Experimental Section

### 3.1 System Description

The developed system was built around the FLAME‐T‐VIS‐NIR‐ES (by Ocean Insight, USA) spectrometer, which is a miniature spectrometer working in the ultraviolet – NIR region of the electromagnetic spectrum. The detector has a range of approximately 345–1,041 nm with a pixel count of 3,648. Fitted with a 25 µm slit, the optical resolution is approximately 0.8 nm. The analog‐to‐digital converter (ADC) resolution is 16 bits. The single‐fiber leg of a reflector/backscatter probe (QR400‐7‐VIS‐BX by Ocean Insight, USA) was attached to the spectrometer, while the other six‐fiber leg intended for a light source was left unattached. A tungsten halogen light source (HL‐2000‐HP by Ocean Insight, USA) with a spectrum range of 360–2,400 nm was tested as a light source, but it was unable to produce an adequate PPG signal. This was possibly due to the very small fiber core size of the probe of only 400 µm as the distance between a light source and a detector affects the signal quality.^[^ ^32^ ^]^

Due to the difficulties with the probe light source, a custom LED printed circuit board (PCB) was designed to function as an external light source. The following LEDs were used in this study: a violet LED (APTR3216‐VFX by Kingbright, Taiwan), a multi‐color LED with blue and green LEDs (APHBM2012LVBDZGKC by Kingbright, Taiwan), and a white LED with a wide spectrum aiming to be close to that of the Sun (S1S0‐3030509503‐0000003S‐0P001 by Seoul Semiconductor Inc, South Korea). An infrared LED (APT1608SF4C‐PRV by Kingbright, Taiwan) was also assembled to the PCB but the signal‐to‐noise ratio was too low. The LEDs were controlled with a constant‐current LED driver (TLC59711 by Texas Instruments, USA).

External pressure was generated using a stepper motor (28BYJ‐48, generic brand) and a threaded shaft in a similar fashion to ref. [15, 33]. The rotation of the threaded shaft slowly lowers a bar, guided by two metal rods at the ends, to a fingertip, causing it to squeeze against the probe‐LED sensor configuration. Similarly, when the rotation of the stepper motor was reversed, the bar was raised to ease pressure between the fingertip and the sensor. A 50‐Newton load cell (FX292X‐100A‐0010‐L by TE Connectivity, USA) was mounted between the stepper motor and the bar to measure the applied force. As the external pressure (pressure of the fingertip against the sensor) nears the mean arterial pressure (MAP) of the large arteries, the arteries start to pulsate maximally, at which point the output of the load cell shows a maximal amplitude in the AC component. The analog voltage produced by the load cell was converted to a digital signal using a 16‐bit ADC (ADS1115 by Texas Instruments, USA).

**Figure 1:**
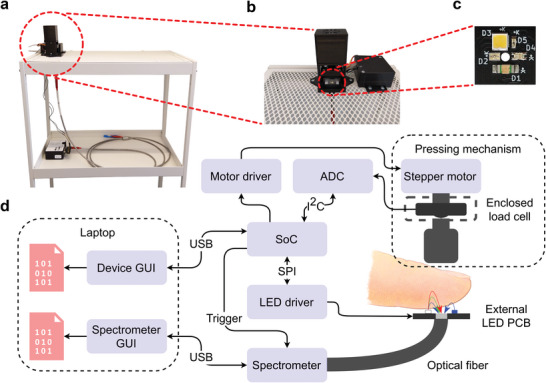
The developed system. a) A picture showing the finger pressing system mounted on top of a trolley with the spectrometer placed below. The spectrometer probe is attached to the bottom of the finger pressing device. b) The finger pressing system pictured from the front. The small box on the right contains the controlling electronics. c) A close‐up picture of the LED PCB with the following silkscreen markings: D1 = violet LED, D3 = white LED, and D4 = a dual‐color LED with blue and green LEDs. The D2 and D5 LEDs were not used in this study. The PCB has a small hole in the center to allow light passage into the optical fiber of the spectrometer probe. That is, the spectrometer probe is attached to the bottom of the LED PCB so that the optical fiber is aligned with the hole of the PCB. The thickness of the PCB is just 0.4 mm (about 1.4 mm with the LEDs). d) System block diagram of the developed system.

All components were controlled by a system‐on‐chip (SoC) (ESP32‐S3 by Espressif Systems, China). The SoC was also connected to the spectrometer using a breakout PCB (HR4‐BREAKOUT by Ocean Insight, USA) that separates the signals from the DD4 (40‐pin) connector of the spectrometer. This connection was used to provide an external trigger signal for the spectrometer to synchronize its data sampling with the rest of the system. A system block diagram of the developed system is presented in **Figure** **1**.

The PCB was designed with the Autodesk Eagle 9.6.2 program (USA), while the plastic parts of the system were designed using the Autodesk Inventor Professional 2022 program (USA). The parts were manufactured using the fused filament fabrication 3D printing technique (Creality CR‐20 Pro, China) with PLA (polylactic acid) filament.

### 3.2 Software

The SoC was programmed in the C programming language using the Espressif internet‐of‐things development framework (ESP‐IDF) version 4.4.1. The firmware was based on two tasks, the first listening for commands from a graphical user interface programmed in Python specifically for the system, and the second one controlling the stepper motor. Timer interrupts were used to sample the load cell and trigger the spectrometer. The sampling rate was 50 Hz. The load cell reading, stepper motor state, and timestamps were sent to the computer over serial connection. Software developed by the spectrometer manufacturer (OceanView 2.0.12) was used to acquire and store the spectra for later analysis.

### 3.3 Signal Processing

#### 3.3.1 PPG Signal Extraction

The data file stored by the software of the spectrometer was a text file where each column has the ADC value of a single pixel (mapping to a specific wavelength of light) of the charge‐coupled device (CCD) sensor. Thus, each row in the file represents a captured spectrum at a certain point in time, while each column was effectively a unique PPG channel corresponding to a specific wavelength of light. Therefore, ideally, one could obtain 3,648 PPG channels from a single measurement. However, during the initial testing of the system, it was noticed that the PPG signals extracted from the raw spectrometer data were rather noisy. Therefore, wavelength binning, where adjacent pixels were averaged, was applied to the raw spectra to improve the signals.

#### 3.3.2 Oscillometry

**Figure 2:**
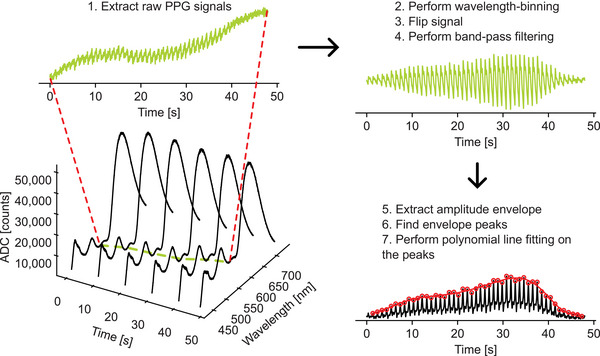
Signal processing pipeline for extracting oscillograms from raw spectra. The external pressure level corresponding to the maximum of the envelope is the MOP.

The process of extracting an oscillogram from a raw wavelength‐binned PPG signal involved the following steps: i) flip the PPG signal so that the systolic peaks point up, ii) filter the signal with a 4th order zero‐phase Butterworth bandpass filter with cutoff frequencies set to [0.5, 8.0], iii) extract the amplitude envelope by computing the absolute value of the Hilbert transform, iv) find the peaks from the resulting amplitude envelope with the automatic multiscale‐based peak detection (AMPD) algorithm,^[^ ^34^ ^]^ and v) calculate an 11^th^‐order polynomial fit on the amplitude envelope peaks to extract the final oscillogram envelope. To avoid poor fittings, each measurement was manually investigated to find the beginning and end of the oscillogram. The signal processing pipeline is illustrated in **Figure** **2**. The level of external pressure corresponding to the maximum of the resulting envelope was defined as the MOP. External pressure levels were calculated from the DC component (extracted with a 4^th^ order zero‐phase Butterworth lowpass filter with a cutoff frequency set to 0.5 Hz) of the load cell signal.

**Figure 3:**
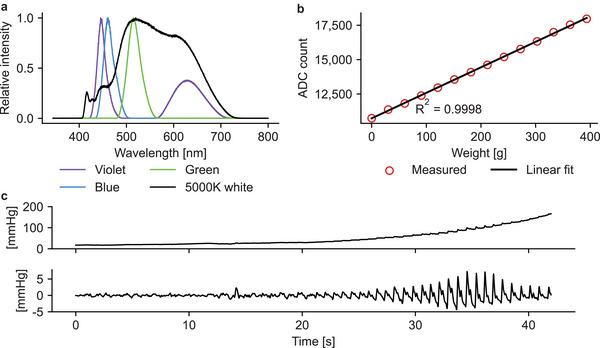
Characteristics of the developed device. a) Spectra of the LEDs relative to the measured peak wavelength. b) Linearity of the load cell. c) An example of the load cell signal during an oscillometric measurement. The top panel shows the raw signal, whereas the lower panel shows the extracted AC component.

The ADC readings of the load cell were converted to millimeters of mercury (mmHg) by finding the ADC reading that corresponds to the MOP of the load cell signal (obtained using the aforementioned steps ii–v)) and then relating it to the MAP of the reference device. Considering that the response of the load cell was linear (see **Figure** **3**b), the ADC readings were converted to mmHg using the equation:

(1) $$P_{ext}=\frac{MAP_{ref}}{s_{lc}^{MOP}-s_{lc}^{0}}\left(s_{lc}-s_{lc}^{0}\right)$$

where *P*_*ext*_ is the external pressure signal in mmHg, *MAP*_*ref*_ is the MAP (in mmHg) of the reference device, $s_{lc}^{MOP}$ is the load cell signal value at MOP (in ADC counts), $s_{lc}^{0}$ is the load cell value without any load (in ADC counts), and *s*_*lc*_ is the load cell signal (in ADC counts).

#### 3.3.3 Perfusion Index

PI was calculated as the ratio of PPG waveform amplitude to DC level. Waveform amplitude was calculated by subtracting a waveform foot value from a waveform peak value. The feet and peaks were extracted from the AC component using the AMPD algorithm. The amplitude was then divided by the DC level at the location of the foot.

#### 3.3.4 Area Under Curve

AUC_lp_ was calculated as follows:

1. Flip the raw PPG signal so that the systolic peaks point upward. The relationship between the raw PPG signal and the blood volume is inverse: the raw PPG signal decreases when the blood volume increases (light absorption due to blood increases, and thus less light is detected by the photodetector) and vice versa.
2. Apply a 4^th^ order zero‐phase Butterworth lowpass filter with a cutoff frequency of 8 Hz. The filtered signal thus consists of the DC and AC components of a PPG signal without high‐frequency noise. The DC component is a proxy for the average blood volume, while the AC component represents the pulsatile blood volume.
3. Normalize the lowpass‐filtered signal to the range [0, 1]. This removes the variation in light intensity between the channels.
4. Compute the area under the PPG signal that approximates the total volume of blood probed.
5. After calculating the area for each PPG channel, normalize the values to the range [0, 1]. This eliminates individual variation between study subjects.

### 3.4 Human Studies

The developed device was tested on ten subjects (four women, *mean* ± *standard deviation* age of 40 ± 14 years) in a sitting position with a reference BP monitor (Omron M3, HEM‐7154‐E) attached to the upper arm and the index finger of the left hand positioned above the spectrometer probe and the external LEDs. Subjects were asked to gently lift the fingertip against the pressing mechanism above the fingertip. The pressing mechanism was then gradually lowered with the fingertip slowly squeezing against the sensing setup and the surrounding flat device structure. The direction of the pressing mechanism was reversed above the level of SBP. Thus, the measurements were based on the oscillometric technique in which the external pressure is gradually either increased above the SBP or lowered from a pressure level above the SBP. Oscillometry is a standard technique in arm cuff‐based automatic BP monitors, and the research group has demonstrated the tonometric version of the technique used in this study in ref. [15, 33]. Note that the proposed system records both pressure‐related oscillations, detected by the load cell, and volumetric oscillations, detected by the spectrometer.

The *mean* ± *standard deviation* SBP, MAP, diastolic BP (DBP), and heart rate readings recorded with the reference device were 121 ± 9 mmHg, 92 ± 5 mmHg, 78 ± 4 mmHg, and 75 ± 12 bpm, respectively. The MAP was estimated using the simple equation: *MAP* = 1/3*SBP* + 2/3*DBP*.^[^ ^35^ ^]^ The study (identifier name OPSENS) was approved by the Ethics Committee of the University of Turku and was conducted according to the Declaration of Helsinki guidelines. Written informed consent was obtained from all study subjects.

### 3.5 Statistical Analysis

The dataset has been described in Section 3.4. Signal processing, including preprocessing and parameter calculations, has been detailed in Section 3.3. Data presentation has been detailed under each figure. Data analysis was performed using Python.

## 4 Results

### 4.1 System Characterization

Figure 3a shows the spectra of the LEDs used. The white LED, being labeled as having an emission spectrum resembling that of the sunlight, indeed has a rather wide and smooth spectrum in contrast to the more typical white LED spectrum with a distinct large spike in the spectrum corresponding to blue light. The violet, blue, and green LEDs compensate for the white LED in the shorter end of the spectrum.

Linearity and drift of the load cell were measured to guarantee the suitability of the load cell for measuring contact pressure. The linearity measured was excellent, as shown in Figure 3b, which depicts the response to the addition of weights on top of the load cell. Additionally, the drift of the load cell was negligible, only ‐1.1% over a period of two minutes with approximately 400 g of weight stacked on top of the load cell. Figure 3c shows an example of an oscillometric measurement in which the external pressure gradually increased above the SBP. The quality of the recorded signal is high, making it easy to extract the level of external pressure corresponding to the maximum oscillation amplitude. Thus, the load cell can record good‐quality signals regardless of the rather inconvenient location above the fingertip.

**Figure 4:**
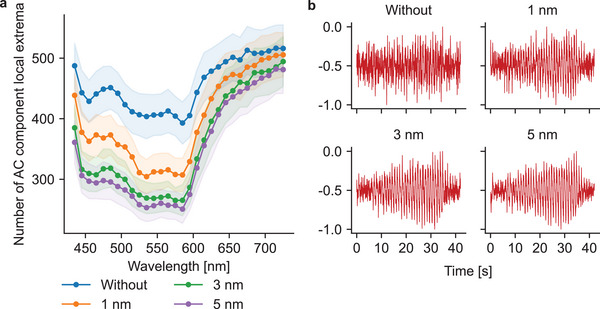
Wavelength binning in PPG signal extraction. a) Number of local extrema (both minimum and maximum) in the PPG AC components. The lines mark the mean values on the whole data set, whereas the shaded areas around the lines mark the ± standard errors. The number of local extrema decreases with increasing wavelength‐binning, which is a sign that the AC components become less noisy. b) An example of wavelength‐binning at a center wavelength of 685 nm (red light) in one measurement. Without any binning, the oscillometric shape of the AC component is very hard to see. Already with a 1 nm wide binning, the shape is easier to detect and with a 3 nm wide binning the shape is clear (maximum amplitude occurs around 35 s). The difference between a 3 nm wide binning and a 5 nm wide one is rather small.

The effect of wavelength binning is demonstrated in **Figure** **4**a, which shows the number of local extrema in a PPG AC component without binning and with a binning of 1, 3, and 5 nm. The number of local extrema works as a simple measure of noise, as noisy signals have more abrupt spikes and turns than clean signals. The effect of binning is clear throughout the spectrum, but especially for channels below 600 nm. Although the effect does not appear to be as significant at the longer end of the spectrum, in practice it can have a significant effect in obtaining an oscillogram from a signal, as demonstrated in Figure 4b. Without binning the oscillometric response is barely visible but already with a 1 nm wide binning the response is more notable, i.e., the amplitude maximum clearly occurs at approximately 35 s. The difference between a 3 nm and a 5 nm wide binning is not large, as shown by both figures, and thus in this study a 3 nm wide binning was used to obtain a larger number of PPG channels. The spectrum covered is approximately 433.5–730.5 nm with center wavelengths of approximately 435, 438, 441,…, 723, 726, 729 nm. That is, the first PPG channel covers the spectrum from approximately 433.5 to 436.5 nm with a center wavelength of approximately 435 nm. In total, the number of PPG channels is 99.

### 4.2 Human Studies

**Figure 5:**
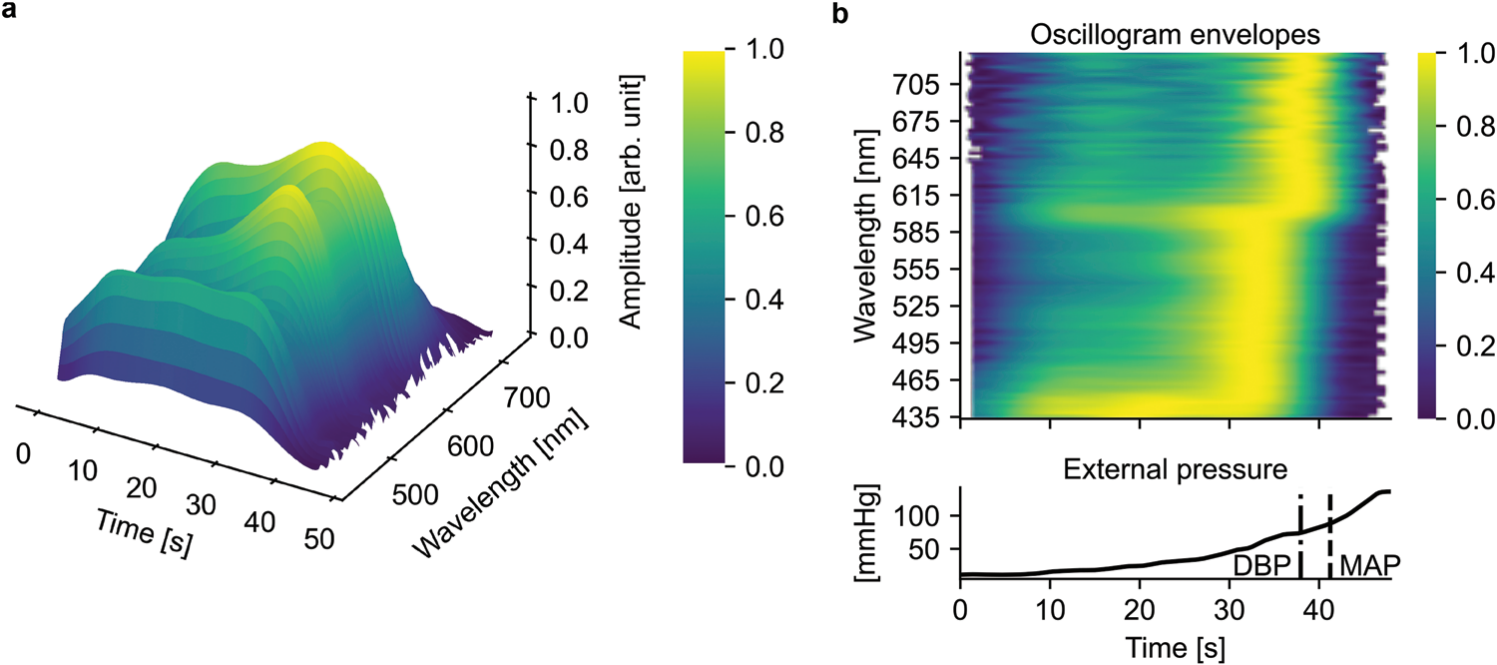
An example of a measurement performed with the developed device. a) A 3D plot of polynomial fittings, normalized to the range of [0, 1] over all wavelengths. b) The same measurement as in the Figure 5a but as a spatial sensitivity profile with row‐wise (wavelength‐wise) [0, 1] normalization. Note that the top (oscillogram envelopes) and bottom (external pressure) figures share the same x‐axis (time axis).

An example of an oscillometric measurement is shown in **Figure** **5**a. The figure displays the extracted oscillogram envelopes as a 3D surface plot. The oscillograms at the shorter end of the spectrum are clearly different from those at the longer end, with flatter envelopes and less distinct peaks. The same data are presented as a spatial sensitivity map in Figure 5b with the data normalized to the range [0, 1] along the wavelength axis. The flatness of the shorter end is clearly visible. The envelope maxima occur approximately at the same external pressure level between 450–585 nm. Approximately at 600 nm, the maxima shift to a higher external pressure level. This phenomenon is common to all measurements, as illustrated in the Supporting Information, where figures similar to Figure 5 are presented for all measurements.

**Figure 6:**
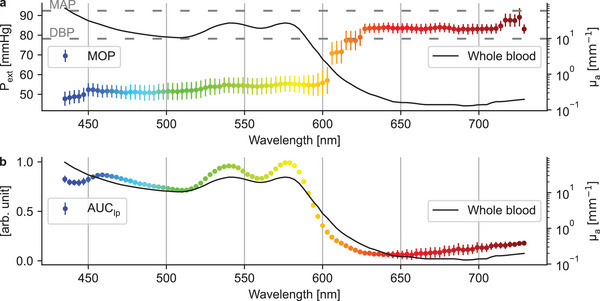
Results on MOPs and AUC_lp_ as *mean* ± *standard error*, calculated over the entire dataset. a) MOPs on the left axis and the absorption spectrum of whole blood on the right axis. The individual MOP curves were filtered with a median filter (window size of three) to remove spikes in the red part of the spectrum before computing the mean curve. The spikes were caused by incorrectly detected oscillogram envelope maxima. The absorption coefficient (µ_*a*_) of whole blood (SO_2_ > 98%) as a function of wavelength (i.e., the absorption spectrum) is based on ref. [36]. b) Lowpass‐filtered PPG areas, AUC_lp_, on the left axis, and the absorption spectrum of whole blood, on the right axis.

**Figure 7:**
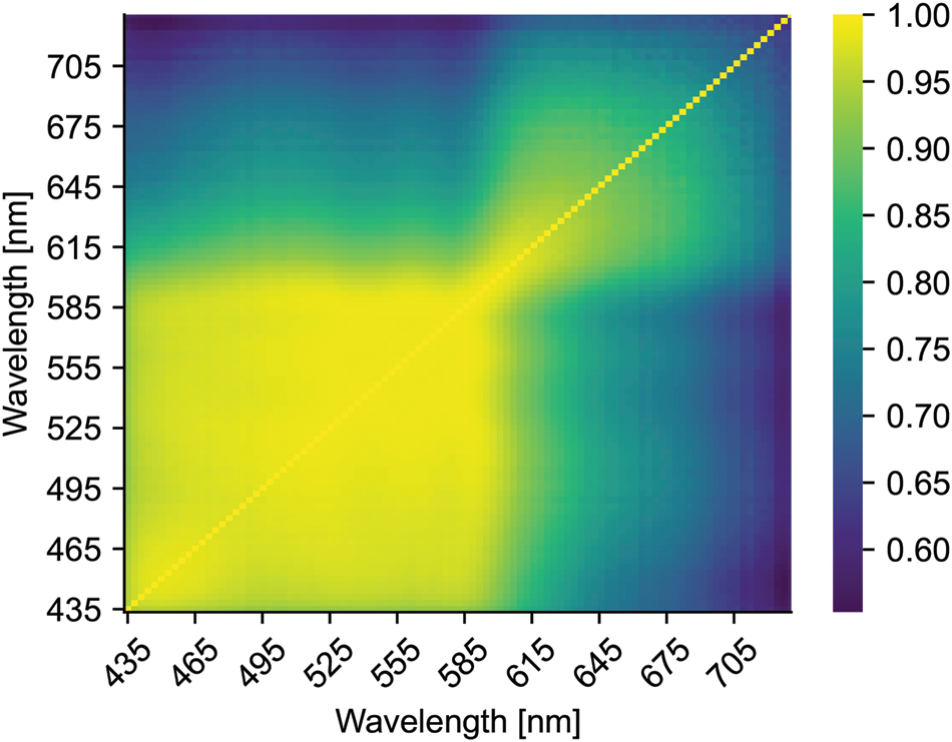
Spatial sensitivity map showing the mean correlations between the AC components of different channels.

**Figure** **6** presents the levels of external pressure at which the envelope maxima, i.e., MOPs, occur for each channel and over the entire dataset. The MOPs show a rather steady increase up to approximately 590 nm, at which point the values, indeed, start to shift to a higher pressure level. After approximately 630 nm, the MOPs stay almost flat, somewhat below the MAP of the reference device. Pearson correlation coefficients between the PPG AC components, from which the MOP values are ultimately extracted, show a similar division in **Figure** **7**. In those results, a clear transition band between 585–615 nm is observed: the channels below the transition band correlate strongly with each other, as do the channels above it, although slightly less strongly.

The sudden increases in MOPs at a point where the blood absorption coefficient decreases rapidly (see Figure 6a) along with the clear changes in the correlation coefficients led to the hypothesis that blood absorption is a major factor affecting oscillometric PPG signals. To test this hypothesis, the AUCs of the lowpass‐filtered, flipped, PPG signals were calculated. These lowpass‐filtered signals are shown for every tenth channel in Figure S11 (Supporting Information). The AUC_lp_ values approximate the total blood volumes (both non‐pulsatile and pulsatile) probed during the measurements, considering that PPG measures variations in blood volume. Therefore, it is reasonable to assume that the AUC_lp_ values reflect the absorption spectrum of whole blood. Figure 6b was obtained by removing the intensity variation between the PPG channels and the variation between individuals. To a slight surprise, the resulting figure very strongly follows the absorption spectrum, especially from approximately 450 nm onward, with, for example, distinct peaks matching those in the absorption spectrum at approximately 540 and 575 nm.

**Figure 8:**
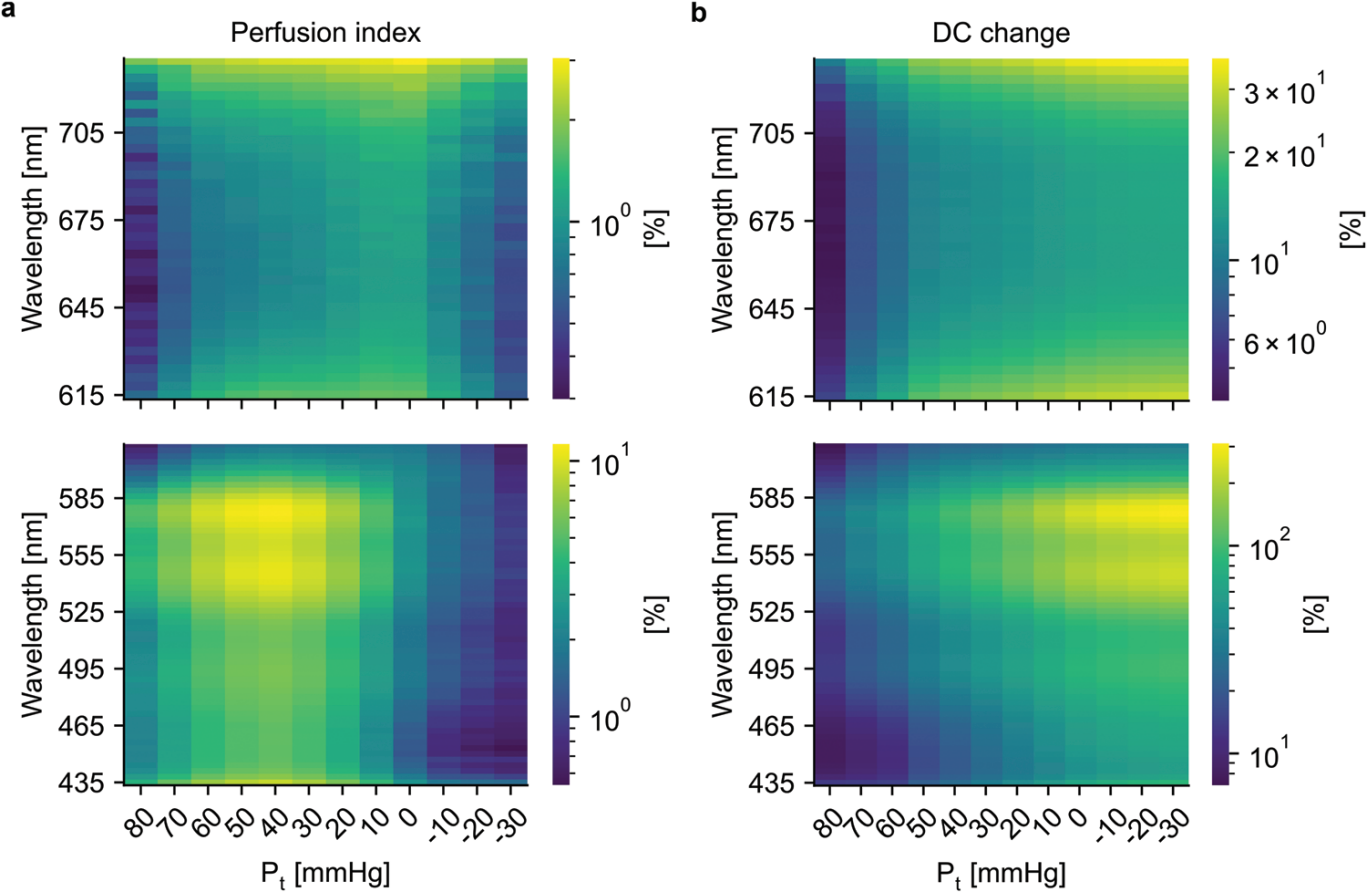
Results on PI values and DC components. a) Mean PI values calculated over the whole dataset as a function of P_t_. The values have been calculated for 10 mmHg wide P_t_ bins. The left panel contains wavelengths below 615 nm, and the right panel contains wavelengths at or above 615 nm. b) Mean DC component level changes calculated over the whole dataset as a function of P_t_. The results have been presented in a similar fashion to the figure (a).

Taking into account the clear division in the above results, PIs and DC component levels were investigated from the perspective of two channel groups separated at 615 nm, an approximate midpoint in the transition band. The calculated PI values are presented in **Figure** **8**a as spatial sensitivity maps with respect to wavelength and transmural pressure defined as: P_t_ = P_inside_ ‐ P_outside_, where P_inside_ is the BP inside the blood vessel (assumed to be equal to MAP) and P_outside_ is the pressure outside the blood vessel, i.e., the external pressure. The PI is clearly dominated by the AC component considering that below 615 nm the maximum values occur around P_t_ = 40 mmHg (mean MAP of 92 mmHg minus an approximate MOP of 54 mmHg in the Figure 6a) and above 615 nm around P_t_ = 10 mmHg (92 mmHg minus an approximate MOP of 83 in the Figure 6a). An additional proof of this is that the channels below 615 nm have PI values close to zero in the negative P_t_ region. This is because the AC components show little to no pulsation at such high external pressure levels, as the more superficial, lower‐pressure, blood vessels probed by the channels have already occluded under the high external pressure. Overall, the highest PI values are obtained for channels between 525–585 nm (corresponding to green and yellow light), with the higher end of this range having slightly higher values. The channels below and especially above the band have significantly lower PI values.

Figure 8b shows similar spatial sensitivity maps for the DC component level changes. The division of the channels is visible here as well, with channels below 615 nm showing significantly greater changes than channels above 615 nm. Similarly to the PI values, the highest values are clearly obtained for the channels between 525–585 nm.

## 5 Discussion

We devised a non‐invasive system capable of obtaining high‐resolution optical signals from the fingertip under varying external pressure. The recorded multi‐wavelength signals and the extracted parameters, MOP, PI and DC component level, showed clear differences as a function of wavelength, implying differences in the contribution of the smallest blood vessels (microcirculation) and the large arteries. The system was developed on the basis of i) the relationship between the wavelength of light and probing depth and ii) the response of the tissue to external pressure. External pressure was chosen as the modifiable parameter as it has a clear effect on tissue response. Too high contact pressure can block blood circulation, whereas an optimal contact pressure close to the mean BP of the blood vessels probed by the given wavelength provides maximal pulsation. Different blood vessels have different pressures, and therefore a pressure sweep similar to what is used in oscillometric measurement was used.

The results on MOPs showed that the channels can be divided into three categories: i) channels below 590 nm, ii) transition band channels between 590 and 630 nm, and iii) channels above 630 nm. The increase in MOPs from category i) to iii) shows that the wavelength of light defines what type of blood vessels are probed the most. In our previous study^[^ ^15^ ^]^ we obtained similar results with a five‐channel LED system, and similar observations have also been presented in ref. [37]. However, the increased spectral resolution in this study showed that the differences in MOPs are rather limited in categories i) and iii), and that there is a clear jump in MOPs in category ii). Additionally, in our previous study,^[^ ^15^ ^]^ the behavior of yellow light was found to be clearly different from the other four channels, with a tendency to show two‐peak oscillograms. Considering the presented results and the wavelength used in the previous study (590 nm, i.e., just within the transition band), we believe that the transition band channels (category ii) are more affected by inter‐individual differences and possibly intra‐individual changes (e.g., in vascular tone).

The range of wavelengths of approximately 600 to 1,300 nm is often described as an optical “window” because the absorption coefficients of the main absorbing components within the skin (for example, HbO_2_, Hb, bilirubin, and melanin) are significantly reduced compared to the shorter end of the spectrum.^[^ ^16^ ^]^ Thus, the skin and blood circulating in the vessels suddenly become optically more transparent, allowing light to penetrate deeper into the skin, and thus probe high‐pressure (large) arteries. That is, a shift from probing more superficial small blood vessels to probing deeper large arteries occurs around 600 nm, which explains the dramatic increase in MOPs. A similar conclusion of an abrupt change in penetration depth was drawn in ref. [22], where the PPG phase shift was observed to change radically at around 600 nm.

The AUC_lp_ results showed that in oscillometric optical measurements, the area under the inverted PPG signals closely matches the absorption spectrum of 98% oxygenated whole blood (i.e., mainly HbO_2_). The lowpass‐filtered PPG signals in Figure S11 (Supporting Information) show that these areas are dominated by the DC component, which relates to tissues and average blood volume.^[^ ^9^ ^]^ Given the clear relationship between AUC_lp_ and blood absorption coefficient, it is evident that the DC component and thus AUC_lp_ are dominated by average blood volume. The initial rapid decrease in the DC component in the longer wavelength range has been thought to be associated with the emptying of the low pressure system, while the rest of the signal, with a fairly stable part before another decrease to the point of full occlusion, is explained by the gradual emptying of the arterial vessels.^[^ ^38^ ^]^ In the shorter wavelength range (wavelengths approximately below 590 nm), less pronounced decreases are observed during the emptying of the low‐pressure vessels. At the very short end (approximately below 460 nm), AUC_lp_ shows a notch, approximately at the same point where the absorption spectrum of deoxygenated whole blood increases, as shown in Figure S12 (Supporting Information). This could imply a greater contribution of the venous blood at the very short end of the investigated spectrum.

Yellow (≈580 nm) and green (≈540 nm) light had the highest PI, indicating the highest ratios of pulsatile blood volume to non‐pulsatile blood volume, represented by the DC component. Therefore, these wavelengths are the most optimal for assessing local tissue perfusion. The renewed interest in PI in the fields of anesthesia, perioperative, and critical care^[^ ^7^ ^]^ should therefore pay attention to the wavelength of light used and the contact pressure of the PPG sensor, as they clearly impact the results. Deriving robust PI‐based indicators would still be difficult, or at least they would be sensitive to device construction (e.g., sensor geometry).

The results on DC components further highlighted that green and yellow light are the most sensitive to average blood volume, suggesting their potential as optimal wavelengths to monitor vascular tone. In fact, in our previous study^[^ ^15^ ^]^ we found that the DC component of yellow PPG reacted the most to pressure‐induced vasodilation, and in ref. [39] we showed that the DC component of green PPG can be used to compensate for vasomotor activity in a continuous BP monitoring system. A somewhat similar conclusion was made in ref. [11] where yellow light was used as a proxy for the arteriolar signal – arterioles are known to play a significant role in vasomotor activity.^[^ ^18^ ^]^ Based on the AUC_lp_ results, the reason for the high PI and DC values for green and yellow light is due to the peaks in the blood absorption spectrum. In addition, these wavelengths have decreased melanin absorption compared to shorter wavelength light, and hence are able to probe deeper, larger blood volume. In conclusion, the green and yellow channels probe a larger volume of blood and react strongly to changes in it.

The greatest challenge in developing the proposed device was to construct a setup to measure the contact pressure between the fingertip and the probe‐LED sensor configuration. Due to the long cylindrical shape of the spectrometer probe, it was not possible to use a cuff or a piston‐based approach as in our previous MWPPG device.^[^ ^15^ ^]^ Although the load cell configuration provided good quality signals, it is not ideal, as there is a mismatch between the surface area covered by a squeezed fingertip and the area of the finger covered by the pressing mechanism above the fingertip. This is likely to cause inaccuracies in measuring the true contact pressure, especially at the shorter end, where the measured MOP values were quite a lot higher than in our previous study (e.g., for 465 nm approximately 51 mmHg vs. 30 mmHg in ref. [15]). Another challenge was to improve the quality of the extracted MWPPG signals. Averaging adjacent wavelengths improved the signals enough for oscillometric analysis, but pulse waveform analysis would not be feasible with the current system, at least for the blue and deep red channels. Finally, in general, MWPPG signals contain overlapping information, considering that the longer‐wavelength channels penetrate through the same superficial layers as the shorter‐wavelength channels. Some model‐based solutions for this have been proposed.^[^ ^12^, ^40^ ^]^ Separation of the information content would lead to improved penetration depth accuracy and thus improved parameter estimation, for example, with respect to MOP.

Future research should investigate the possibility of using yellow light to monitor changes in vascular tone due to the activity of the autonomic nervous system. This could have practical applications, for example, in monitoring sleep cycles in smart watches and smart rings. More research is also needed to investigate the practical use of the MOP curve, for example, in the management of hypertension. This would require performing oscillometric measurements with a system similar to the one presented in this article on a large population including hypotensive, normotensive, and hypertensive subjects to see if the pressure difference between the MOPs of the shorter and longer‐wavelength channels is different in hypertensive patients. If this were the case, it could be a sign of pressure leaking into microvascular vessels (capillary and arteriolar) and could allow indirect measurement of impaired peripheral vascular resistance, a sign of a potential vicious cycle in hypertension.^[^ ^41^ ^]^ Possible differences in the transition band channels could also be of interest. In addition, future studies should also include even shorter wavelengths of light, down to 400 nm, to see if the MOPs decrease further. Based on the absorption spectrum of whole blood,^[^ ^36^ ^]^ this could be possible as the absorption coefficient still increases somewhat below 434 nm.

Overall, the high spectral resolution combined with external pressure control allows the creation of a comprehensive optical monitoring system that minimizes the confounding effects of unregulated sensor contact pressure, providing interesting research opportunities. The high spectral resolution adds a new dimension to the study of various parameters derived from PPG, such as arterial compliance and oxygen saturation (both tissue and peripheral, using short‐end visible light and visible‐NIR, respectively). The signals could also potentially be used to measure different levels of chromophores, such as melanin, hemoglobin, and bilirubin. Bilirubin, for example, is a well‐established marker of liver function and, at high levels, potentially harmful to the brain and nervous system.^[^ ^42^, ^43^ ^]^ With further advances in miniature spectrometric sensors, it becomes possible to create new PPG‐based techniques: Inverting the sensor configuration from narrow‐band LEDs and a wide‐band photodiode to a wide‐band LED and a spectometric detector would maximize the available information content and thus increase the accuracy of current PPG parameters by optimizing, perhaps dynamically, the wavelength and create a wealth of new parameters that could combine shorter‐wavelength (microcirculation‐related) parameters with longer‐wavelength (macrocirculation‐related) parameters. An example of the latter is the arteriolar PTT, calculated using PPG signals recorded at blue, yellow, and infrared wavelengths, recently used in ref. [44] to assess systemic vascular resistance.

## 6 Conclusion

We presented a system that effectively combines the capabilities of spectrometry, PPG, and an arterial pressure measurement device. The system was demonstrated to record MWPPG signals with high spectral resolution from the fingertip. With a built‐in method to control and measure the level of external pressure, we showed that the optical channels divided into three groups as a function of light wavelength, roughly <590 nm, 590–630 nm, and >630 nm, due to the sudden drop in blood absorption coefficient. Based on the significantly lower MOP results, the shorter wavelength (below 590 nm) channels probe smaller and lower‐pressure superficial blood vessels, whereas the longer wavelengths (above 630 nm) probe large arteries deep in the skin. Understanding the difference in the information content of PPG signals recorded at different wavelengths of light and under controlled external pressure could help derive new hemodynamic parameters in the future.

## Conflict of Interest

The authors declare no conflict of interest.

## Author Contributions

J.‐P.S., T.P., and M.K. designed the research; J.‐P.S. developed the device, the software, and analyzed the data; J.‐P.S. performed the research with participation from T.P. and M.K.; M.K., J.‐P.S., and T.P. obtained the funding, and J.‐P.S., T.P., and M.K. wrote the paper.

## Supporting information

Supporting Information

## Data Availability

The data that support the findings of this study are available from the corresponding author upon reasonable request.
