# Supplementary material for: Non‐Invasive Hemodynamic Monitoring System Integrating Spectrometry, Photoplethysmography, and Arterial Pressure Measurement Capabilities
Source: Adv Sci (Weinh). 2024 Apr 22;11(24):2310022. doi: 10.1002/advs.202310022 (PMC11199981; doi:10.1002/advs.202310022)
Supplement: Supplementary file 1 — Supporting Information [file ADVS-11-2310022-s001.pdf]

ADVANCED SCIENCE
Open Access

Supporting Information

for *Adv. Sci.*, DOI 10.1002/advs.202310022

Non-Invasive Hemodynamic Monitoring System Integrating Spectrometry, Photoplethysmography, and Arterial Pressure Measurement Capabilities

Jukka-Pekka Sirkiä, Tuukka Panula and Matti Kaisti**

Supplementary Information – Non-invasive Hemodynamic Monitoring System Integrating Spectrometry, Photoplethysmography and Arterial Pressure Measurement Capabilities

Jukka-Pekka Sirkiä[1,*], Tuukka Panula[1], and Matti Kaisti[1,*]

[1]Department of Computing, University of Turku, Vesilinnantie 5, 20500 Turku, Finland
[*]Corresponding authors: Jukka-Pekka Sirkiä (jpsirk@utu.fi), Matti Kaisti (mkaist@utu.fi)

1 Oscillogram Envelopes

Oscillogram envelopes for each study subject are presented in Figures S1–S10. The subfigures labeled as *(a)* show 3D plots of oscillogram envelope polynomial fittings, normalized to the range of [0, 1] over all wavelengths, for each study subject. The figures labeled as *(b)* show the same data, but as spatial sensitivity profiles with row-wise (wavelength-wise) [0, 1] normalization. Note that the top (oscillogram envelopes) and bottom (external pressure) figures share the same x-axis (time axis).

2 Lowpass-Filtered Photoplethysmography Signals

Lowpass-filtered photoplethysmography (PPG) signals for each study subject are shown in Figure S11. For illustration purposes, only every tenth PPG channel is shown. The computed AUC_{lp} together with the absorption spectra of $SO_2 > 98\%$ and $SO_2 = 0\%$ whole blood (based on [1]) are presented in Figure S12.

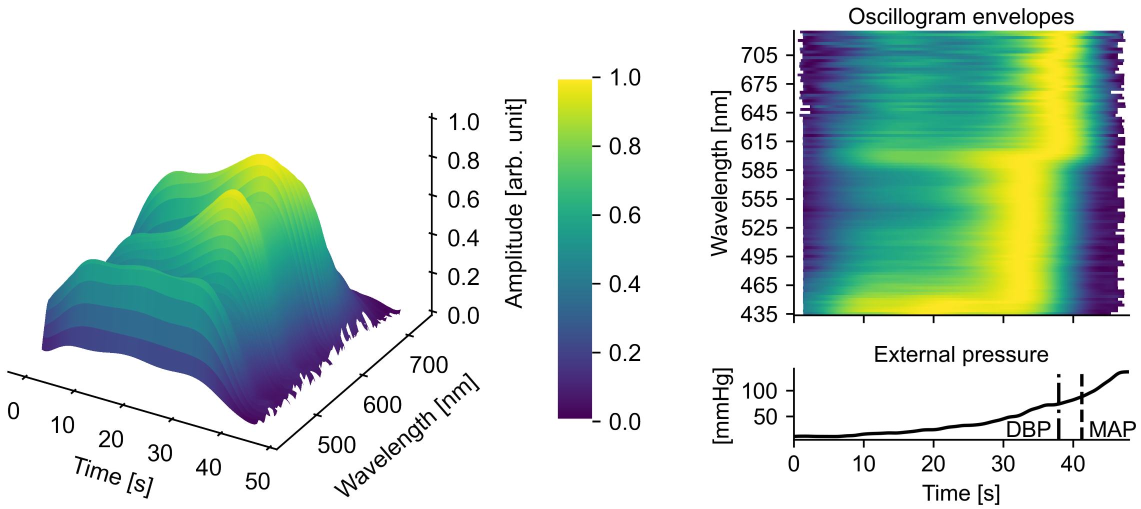

(a) 3D plot of polynomial fittings.

(b) Spatial sensitivity profile of polynomial fittings.

Figure S1: Study subject 1.

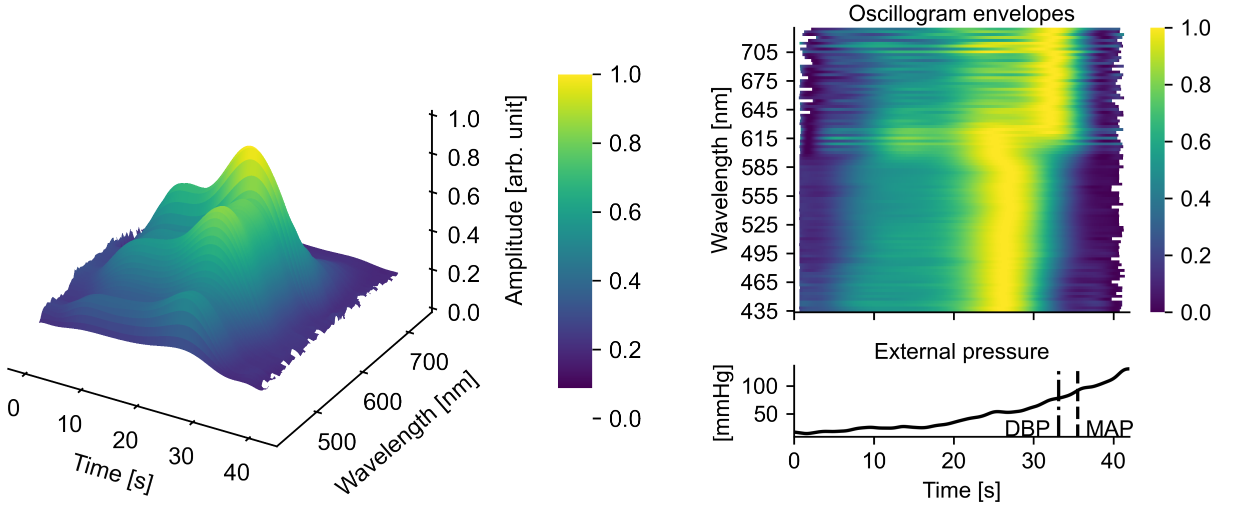

(a) 3D plot of polynomial fittings.

(b) Spatial sensitivity profile of polynomial fittings.

Figure S2: Study subject 2.

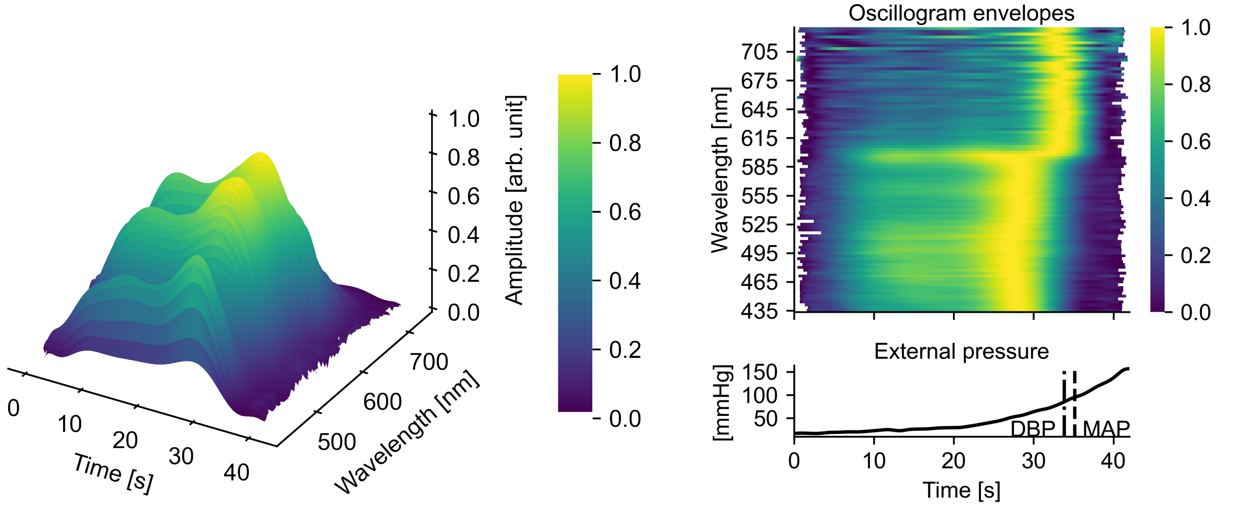

(a) 3D plot of polynomial fittings.

(b) Spatial sensitivity profile of polynomial fittings.

Figure S3: Study subject 3.

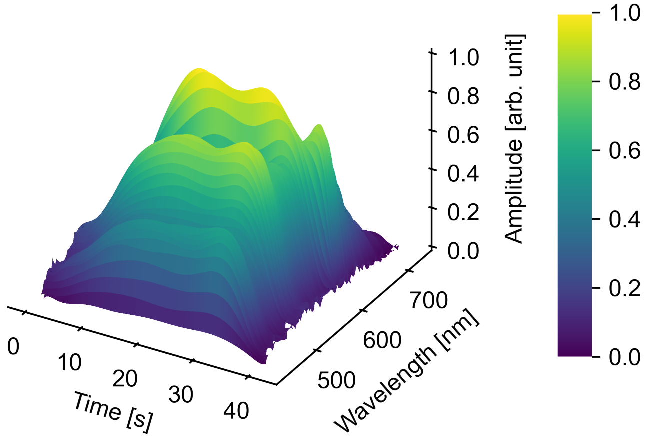

(a) 3D plot of polynomial fittings.

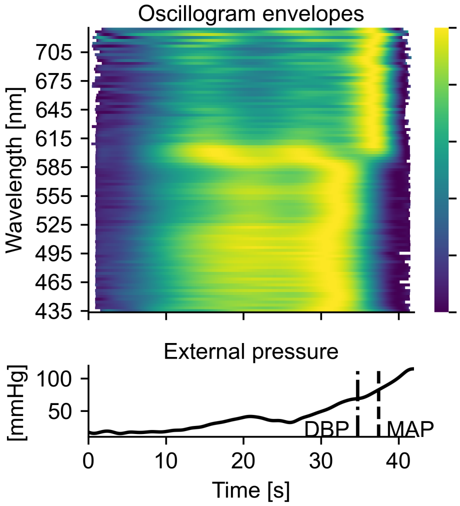

(b) Spatial sensitivity profile of polynomial fittings.

Figure S4: Study subject 4.

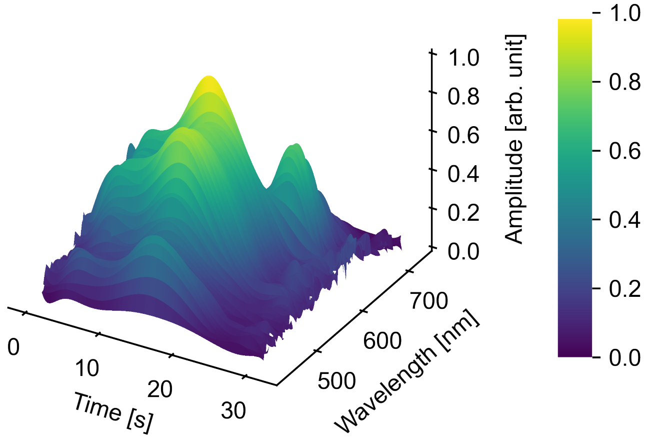

(a) 3D plot of polynomial fittings.

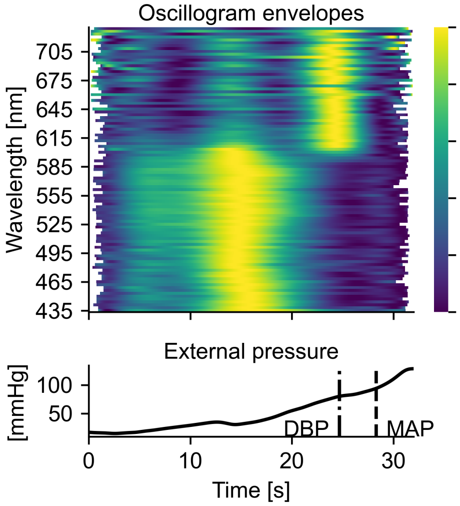

(b) Spatial sensitivity profile of polynomial fittings.

Figure S5: Study subject 5.

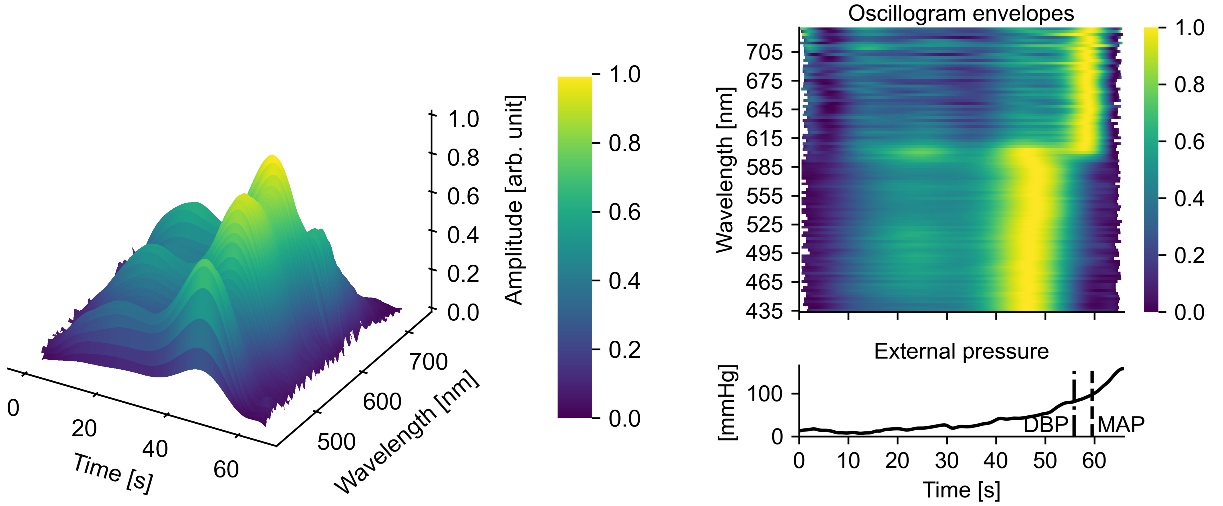

(a) 3D plot of polynomial fittings. (b) Spatial sensitivity profile of polynomial fittings.

Figure S6: Study subject 6.

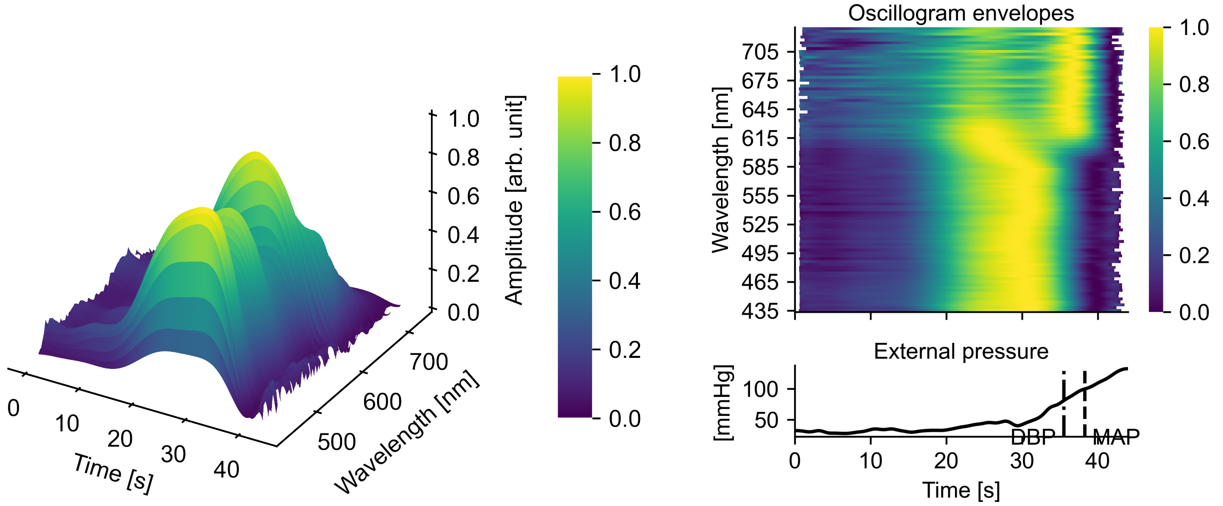

(a) 3D plot of polynomial fittings. (b) Spatial sensitivity profile of polynomial fittings.

Figure S7: Study subject 7.

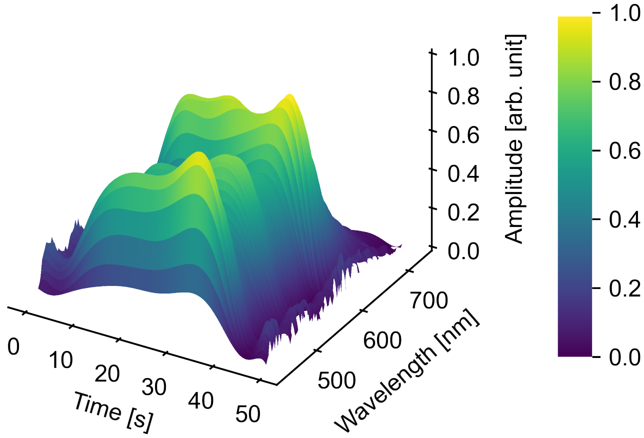

(a) 3D plot of polynomial fittings.

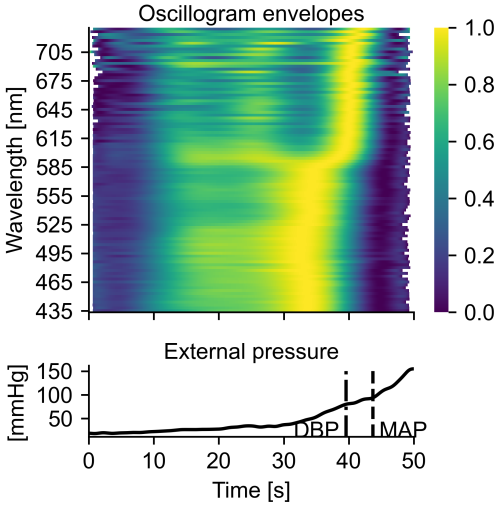

(b) Spatial sensitivity profile of polynomial fittings.

Figure S8: Study subject 8.

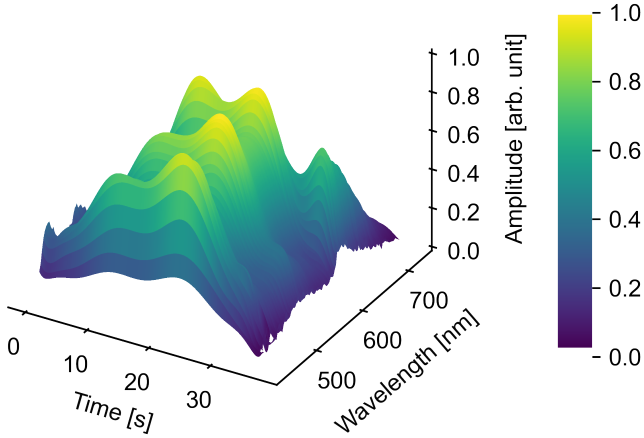

(a) 3D plot of polynomial fittings.

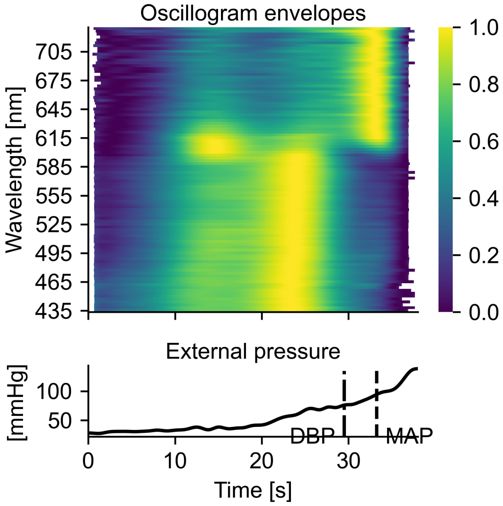

(b) Spatial sensitivity profile of polynomial fittings.

Figure S9: Study subject 9.

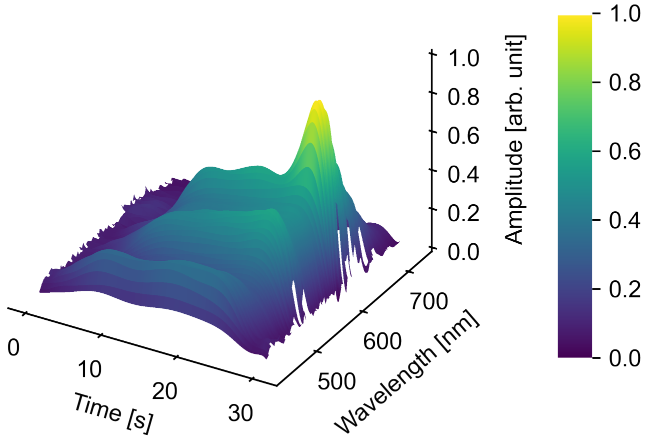

(a) 3D plot of polynomial fittings.

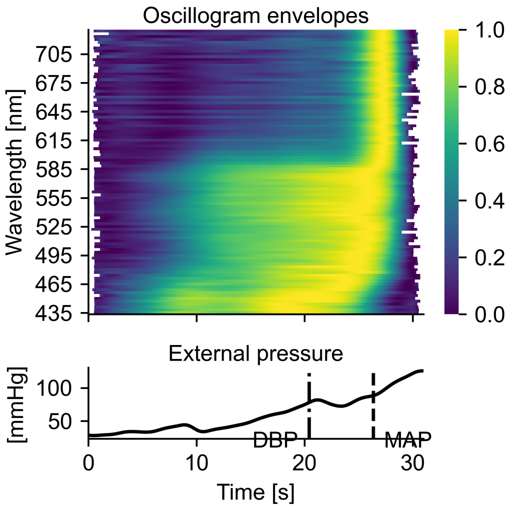

(b) Spatial sensitivity profile of polynomial fittings.

Figure S10: Study subject 10.

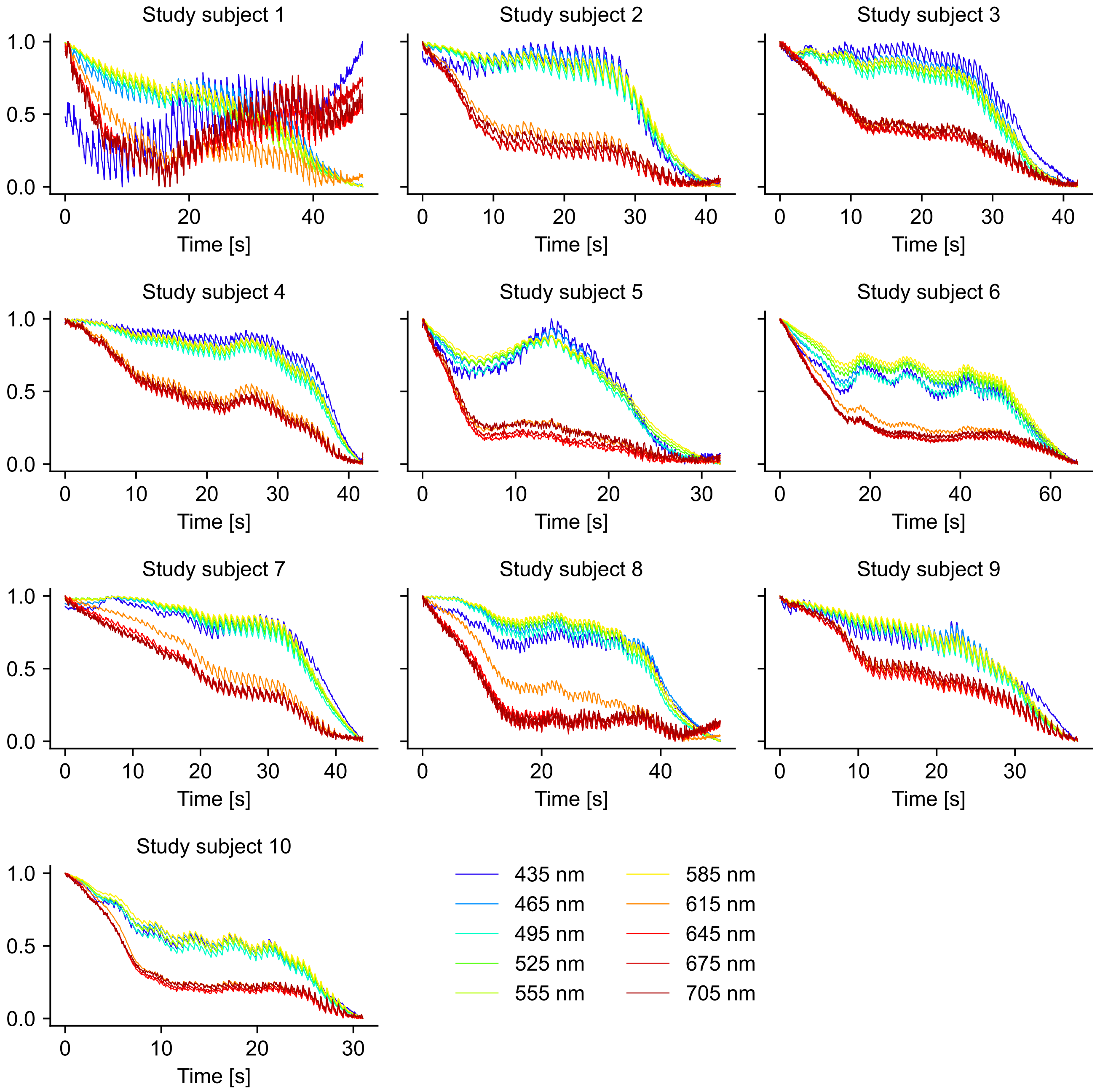

Figure S11: Lowpass-filtered (cutoff frequency of 8 Hz) and [0, 1] normalized PPG signals for each study subject. For clarity, only every tenth PPG signal of the 99 signals was plotted for each subject. As described in the main article, emptying of the low pressure system in the longer-wavelength range (channels above 630 nm) is clear in all figures. For example, for the study subjects 2 and 3 this phase occurs approximately within the first 10 and 13 seconds, respectively.

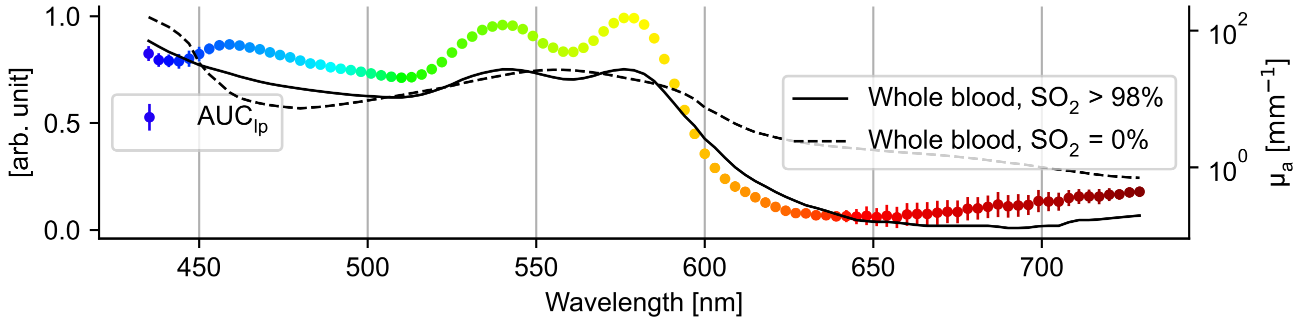

Figure S12: Lowpass-filtered PPG areas, AUC_{lp}, on the left axis, and the absorption spectrum of whole blood, both $SO_2 > 98\%$ and $SO_2 = 0\%$ (based on [1]), on the right axis. The notch in AUC_{lp} below approximately 460 nm occurs around the same point where the absorption coefficient of deoxygenated blood starts to increase.

References

[1] Nienke Bosschaart et al. "A Literature Review and Novel Theoretical Approach on the Optical Properties of Whole Blood". In: *Lasers in Medical Science* 29.2 (Mar. 2014), pp. 453–479. ISSN: 1435-604X. DOI: 10.1007/s10103-013-1446-7.
